# Rumphellols A and B, New Caryophyllene Sesquiterpenoids from a Formosan Gorgonian Coral, *Rumphella antipathies*

**DOI:** 10.3390/ijms150915679

**Published:** 2014-09-04

**Authors:** Hsu-Ming Chung, Wei-Hsien Wang, Tsong-Long Hwang, Jih-Jung Chen, Lee-Shing Fang, Zhi-Hong Wen, Yu-Bao Wang, Yang-Chang Wu, Ping-Jyun Sung

**Affiliations:** 1 Department of Applied Chemistry, National Pingtung University, Pingtung 900, Taiwan; E-Mail: shiuanmin@mail.npue.edu.tw; 2 National Museum of Marine Biology and Aquarium, Pingtung 944, Taiwan; E-Mails: whw@nmmba.gov.tw (W.-H.W.); loveweasel1982@gmail.com (Y.-B.W.); 3 Department of Marine Biotechnology and Resources and Asia-Pacific Ocean Research Center, National Sun Yat-sen University, Kaohsiung 804, Taiwan; E-Mail: wzh@mail.nsysu.edu.tw; 4 Graduate Institute of Natural Products, Chang Gung University, Taoyuan 333, Taiwan; E-Mail: htl@mail.cgu.edu.tw; 5 Department of Pharmacy, Tajen University, Pingtung 907, Taiwan; E-Mail: jjchen@tajen.edu.tw; 6 Department of Sport, Health and Leisure, Cheng Shiu University, Kaohsiung 833, Taiwan; E-Mail: lsfang@csu.edu.tw; 7 School of Pharmacy, College of Pharmacy, China Medical University, Taichung 404, Taiwan; 8 Chinese Medicine Research and Development Center, China Medical University Hospital, Taichung 404, Taiwan; 9 Center for Molecular Medicine, China Medical University Hospital, Taichung 404, Taiwan; 10 Graduate Institute of Marine Biology, Department of Life Science and Institute of Biotechnology, National Dong Hwa University, Pingtung 944, Taiwan; 11 Graduate Institute of Natural Products, Kaohsiung Medical University, Kaohsiung 807, Taiwan

**Keywords:** *Rumphella antipathies*, sesquiterpene, caryophyllene, rumphellol, superoxide anion, elastase

## Abstract

Two new marine-derived caryophyllene-type sesquiterpenoids, rumphellols A and B (**1** and **2**), were obtained from the gorgonian coral, *Rumphella antipathies*, collected off the waters of Taiwan. Although caryophyllene-type sesquiterpenes are rarely found in marine organisms, compounds of this type could be principal components of *R. antipathies*. The structures of new Compounds **1** and **2** were determined by analysis of their spectroscopic data, including 1D and 2D NMR experiments. Caryophyllene **1** and **2** were evaluated in terms of their anti-inflammatory activity by examining their inhibitory effects on the generation of superoxide anions and the release of elastase by human neutrophils.

## 1. Introduction

**Scheme 1:**
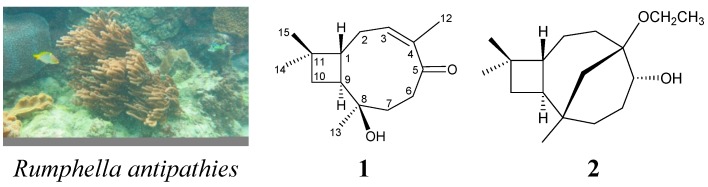
The gorgonian coral *Rumphella antipathies* and the structures of caryophyllene **1** and **2**.

Octocorals, including Alcyonacea and Gorgonacea, have been demonstrated to be rich sources of bioactive natural products [1,2,3,4]. In ongoing studies on the chemical constituents of marine invertebrates collected off the waters of Taiwan at the intersection of the Kuroshio current, the Oyashio current and the South China Sea surface current, organic extracts of the gorgonian coral, *Rumphella antipathies* (phylum Cnidaria, class Anthozoa, order Gorgonacea, suborder Holaxonia, family Gorgoniidae) [5], which is distributed in the tropical waters of the Indo–Pacific Ocean, were studied, and they displayed meaningful signals in NMR studies. Previous studies of *R. antipathies* have yielded a series of interesting caryophyllene- and clovane-type sesquiterpenoids, including rumphellolides A–I [6,7,8,9], rumphellatins A–D [10,11,12], rumphellaone A–C [13,14], kobusone [15], isokobusone [16], rumphellclovanes A–E [17,18,19], 2β-hydroxyclovan-9-one [17], 9α-hydroxyclovan-2-one [18], clovan-2,9-dione [19], 2β-acetoxyclovan-9α-ol [20], 9α-acetoxyclovan-2β-ol [20] and clovan-2β,9β-diol [20]. Compounds of these two types are found in terrestrial plants [21], but are rarely found in marine organisms [22,23,24]. We further isolated two new caryophyllene-type sesquiterpenoids, rumphellols A (**1**) and B (**2**) (Scheme 1), from *R. antipathies*. In this paper, we describe the isolation, structure determination and anti-inflammatory properties of caryophyllene **1** and **2**. Although caryophyllene-type sesquiterpenes are rarely found in marine organisms, compounds of this type could be principal components of *R. antipathies*. Caryophyllene **1** and **2** were evaluated in terms of their anti-inflammatory activity by examining their inhibitory effects on the generation of superoxide anions and the release of elastase by human neutrophils.

## 2. Results and Discussion

Rumphellol A (**1**) was isolated as a colorless oil, and the molecular formula of this compound was determined to be C_15_H_24_O_2_ by high resolution electronspray ionization mass spectrum (HRESIMS) at *m*/*z* 237.1836 (calcd. for C_15_H_24_O_2_ + H, 237.1849). IR absorptions at ν_max_ 3429 (broad) and 1724 cm^−1^ revealed the presence of hydroxy and carbonyl functionalities. The ^13^C NMR spectrum of **1** showed 15 carbon signals (Table 1), which were assigned with the assistance of the distortionless enhancement by polarization transfer (DEPT) spectrum to four methyls, four sp^3^ methylenes, two sp^3^ methines, two sp^3^ quaternary carbons (including an oxygenated quaternary carbon), an sp^2^ methine and two sp^2^ quaternary carbons (including a carbonyl). The ^13^C resonances at δ_C_ 212.7 (C-5) demonstrated the presence of a ketonic carbonyl. From the ^13^C NMR data, a trisubstituted olefin was deduced from the signals at δ_C_ 128.8 (C-3) and 138.4 (C-4). Comparison of the ^13^C NMR and DEPT spectra with the molecular formula indicated that there must be an exchangeable proton, requiring the presence of a hydroxy group. Thus, the NMR data accounted for two degrees of unsaturation and required **1** to be a sesquiterpenoid with two rings. The ^1^H NMR spectrum of **1** (Table 1) showed the presence of four methyl groups, including two methyls attached to a quaternary carbon (H_3_-14 and H_3_-15), a methyl attached to an oxygenated quaternary carbon (H_3_-13) and a vinyl methyl (H_3_-12). In addition, four pairs of aliphatic methylene protons (H_2_-2, H_2_-6, H_2_-7 and H_2_-10), two aliphatic methine protons (H-1 and H-9) and an olefin proton (H-3) were observed in the ^1^H NMR spectrum of **1**.

**Table 1:** ^1^H and ^13^CNMR Data, ^1^H–^1^H correlation spectroscopy (COSY) and heteronuclear multiple-bond coherence (HMBC) correlations for sesquiterpenoid **1**.

C/H	δ_H_ (*J* in Hz)	δ_C_, Multiple	^1^H–^1^H COSY	HMBC (H→C)
1	1.83 m	44.4, CH	H_2_-2, H-9	C-8, -9, -11
2	1.84 m	27.6, CH_2_	H-1, H-3	C-1, -3, -4, -9
	2.20 m			
3	5.64 dd (8.4, 8.4)	128.8, CH	H_2_-2	C-2, -5, -12
4		138.4, C		
5		212.7, C		
6	2.19 ddd (16.0, 12.0, 1.6)	38.0, CH_2_	H_2_-7	C-4, -5, -7, -8
	2.53 ddd (16.0, 8.8, 2.0)			
7	1.88 ddd (12.0, 8.8, 1.6)	37.7, CH_2_	H_2_-6	C-5, -6, -8, -9, -13
	2.03 ddd (12.0, 12.0, 2.0)			
8		72.6, C		
9	1.95 ddd (9.2, 9.2, 9.2)	44.9, CH	H-1, H_2_-10	C-1, -2, -10
10	1.63 dd (10.8, 9.2)	33.6, CH_2_	H-9	C-1, -8, -9, -11, -14, -15
	1.56 dd (10.8, 9.2)			
11		32.8, C		
12	1.82 s	19.8, CH_3_		C-3, -4, -5
13	1.02 s	25.3, CH_3_		C-7, -8, -9
14	0.96 s	29.6, CH_3_		C-1, -10, -11, -15
15	0.96 s	23.2, CH_3_		C-1, -10, -11, -14

**Figure 1:**
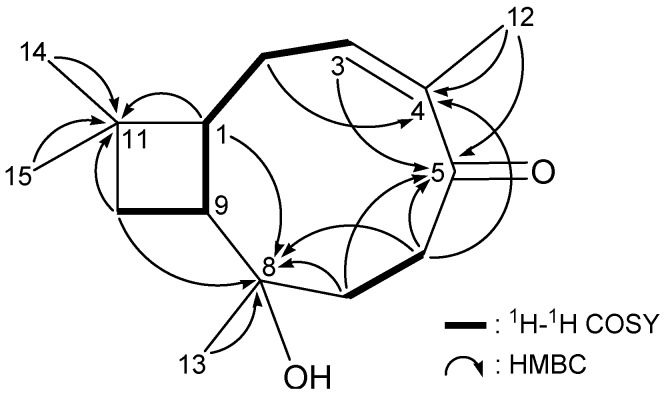
^1^H–^1^H COSY and selective HMBC correlations (protons→quaternary carbons) of **1**.

The gross structure of **1** and all of the ^1^H and ^13^C NMR data associated with the molecule were determined by 2D NMR studies, including ^1^H–^1^H COSY, heteronuclear multiple quantum correlation (HMQC) and HMBC experiments. The ^1^H NMR coupling information in the ^1^H–^1^H COSY spectrum of **1** enabled identification of the C-10/C-9/C-1/C-2/C-3 and C-6/C-7 units (Figure 1). These data (together with the HMBC correlations between H-1/C-8, C-9; H_2_-2/C-1, C-3, C-4, C-9; H-3/C-2, C-5; H_2_-6/C-4, C-5, C-7, C-8; H_2_-7/C-5, C-6, C-8, C-9; and H-9/C-1, C-2 (Table 1 and Figure 1)) established the connectivity from C-1 to C-9 within the nine-membered ring. The methyls attached at C-4 and C-8 were confirmed by the HMBC correlations between H_3_-12/C-3, C-4, C-5 and H_3_-13/C-7, C-8, C-9, respectively. The cyclobutane ring, which is fused to the nine-membered ring at C-1 and C-9, was elucidated by the ^1^H–^1^H COSY correlations between H-9 and H_2_-10 and by the HMBC correlations between H-1/C-11, H-9/C-10 and H_2_-10/C-1, C-8, C-9. These data, together with the HMBC correlations between H_2_-10/C-11, C-14, C-15; H_3_-14/C-1, C-10, C-11, C-15 and H_3_-15/C-1, C-10, C-11, C-14, unambiguously established the planar structure of **1**.

**Figure 2:**
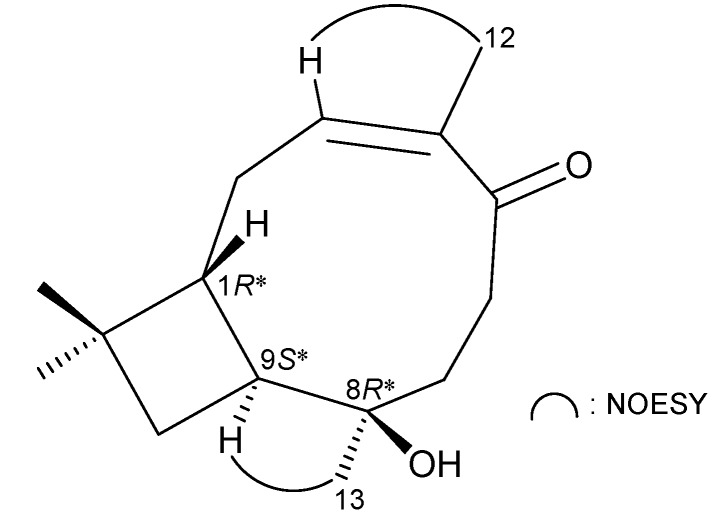
Selective NOESY correlations of **1**.

The stereochemistry of **1** was elucidated from the interactions observed in a nuclear Overhauser effect spectroscopy (NOESY) experiment (Figure 2) and by the vicinal ^1^H–^1^H coupling constants. The *trans* geometries of H-1 and H-9 were indicated by a 9.2-Hz coupling constant between these two ring juncture protons, and H-9 and H-1 were assigned as α- and β-oriented protons, respectively, in **1**. In the NOESY experiment, H-9 exhibited a correlation with H_3_-13, indicating that H-9 and Me-13 are located on the same face and can be assigned as α protons, since H-1 is β-oriented and H-9 did not show a correlation with H-1. Furthermore, H-3 showed an interaction with H_3_-12, revealing the *Z* geometry of the C-3/4 double bond in **1**. Based on the above findings, the configurations of all chiral carbons of **1** were assigned as 1R*, 8R* and 9S*.

Rumphellol B (**2**) was isolated as a colorless oil that gave a pseudomolecular ion [M + Na]^+^ at *m*/*z* 289.2128 in the HRESIMS, indicating the molecular formula C_17_H_30_O_2_ (calcd. for C_17_H_30_O_2_ + Na, 289.2138) and implying three degrees of unsaturation. A broad IR absorption was observed at 3441 cm^−1^, suggesting the presence of a hydroxy group in **2**. The ^13^C NMR and DEPT spectra of **2** (Table 2) showed 17 carbons, including four methyls, seven sp^3^ methylenes (including an oxymethylene), three sp^3^ methines (including an oxymethine) and three quaternary carbons (including an oxygenated quaternary carbon).

**Figure 3:**
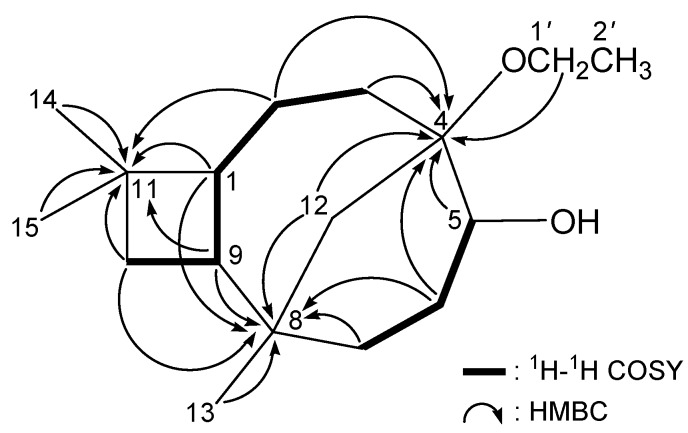
^1^H–^1^H COSY and selective HMBC correlations (protons→quaternary carbons) of **2**.

From the ^1^H–^1^H COSY experiment of **2** (Table 2 and Figure 3), it was possible to establish the spin system that mapped out the proton sequences from H_2_-10/H-9/H-1/H_2_-2/H_2_-3 and H-5/H_2_-6/H_2_-7, which were assembled with the assistance of an HMBC experiment (Table 2 and Figure 3). The HMBC correlations between protons and quaternary carbons of **2** (such as H-1/C-8, C-11; H_2_-2/C-4, C-11; H_2_-3/C-4; H-5/C-4; H_2_-6/C-4, C-8; H_2_-7/C-8; H-9/C-8, C-11; H_2_-10/C-8, C-11; H_2_-12/C-4, C-8; H_3_-13/C-8; H_3_-14/C-11; and H_3_-15/C-11) permitted elucidation of the main carbon skeleton. The tertiary methyl at C-8 was confirmed by the HMBC correlations between H_3_-13/C-7, C-8, C-9, C-12. Moreover, two tertiary methyls at C-11 were elucidated by the HMBC correlations between H_3_-14/C-1, C-10, C-11, C-15 and H_3_-15/C-1, C-10, C-11, C-14. The location of an ethoxy group in **2** was confirmed by the HMBC correlations between the oxymethylene protons (δ_H_ 3.42 and 3.49) and the C-4 oxygenated quaternary carbon (δ_C_ 80.2).

**Table 2:** ^1^H and ^13^C NMR data, ^1^H–^1^H COSY and HMBC correlations for sesquiterpenoid **2**.

C/H	δ_H_ (*J* in Hz)	δ_C_^b^	^1^H–^1^H COSY	HMBC (H→C)
1	1.73 m	44.0, CH	H_2_-2, H-9	C-2, -8, -9, -10, -11, -14, -15
2	1.69 m	21.7, CH_2_	H-1, H_2_-3	C-1, -3, -4, -11
	1.53 m			
3	1.91 m	29.3, CH_2_	H_2_-2	C-1, -2, -4, -5
	1.56 m			
4		80.2, C		
5	3.57 dd (11.2, 6.0)	76.8, CH	H_2_-6	C-3, -4, -6
6	1.61–1.80 m	27.1, CH_2_	H-5, H_2_-7	C-4, -5, -7, -8
7	1.22 ddd (13.2, 13.2, 5.2)	36.6, CH_2_	H_2_-6	C-5, -6, -8, -9, -12
	1.38 dddd (13.2, 4.4, 2.8, 2.8)			
8		32.8, C		
9	2.08 ddd (11.6, 10.0, 8.0)	36.5, CH	H-1, H_2_-10	C-1, -2, -7, -8, -11, -13
10	1.28 dd (10.0, 9.6)	35.5, CH_2_	H-9	C-1, -8, -9, -11, -14, -15
	1.46 dd (9.6, 8.0)			
11		34.9, C		
12	0.92 d (12.8)	42.7, CH_2_		C-3, -4, -5, -7, -8, -9
	1.88 d (12.8)			
13	0.80 s	26.2, CH_3_		C-7, -8, -9, -12
14	0.98 s	30.7, CH_3_		C-1, -10, -11, -15
15	0.97 s	20.8, CH_3_		C-1, -10, -11, -14
4-OEt	3.42 dq (8.8, 7.2)	56.3, CH_2_	H_3_-2'	C-4, -2'
	3.49 dq (8.8, 7.2)			
	1.13 t (7.2)	16.4 CH_3_	H_2_-1'	C-1'

**Figure 4:**
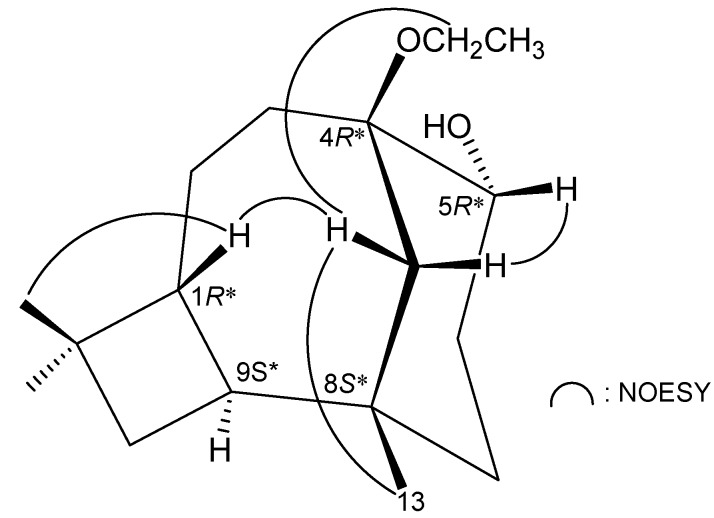
Selective NOESY correlations of **2**.

The relative configuration of **2** was established by an analysis of interactions that were found in the NOESY experiment (Figure 4) and by the vicinal ^1^H–^1^H coupling constants. Due to the α-orientation of H-9, a large coupling constant was found between H-9 and H-1 (*J* = 11.6 Hz), indicating that H-1 has a β-orientation. One of the methylene protons at C-12 (δ_H_ 1.88) exhibited correlations with H-1, H_3_-13 and the oxymethylene protons of the ethoxy group, while the other (δ_H_ 0.92) was correlated with H-5, indicating that the methyl group at C-8 and the ethoxy group at C-4 should be positioned on the equatorial directions in the cyclohexane ring. Based on the above findings, the structure of **2** was established, and the chiral carbons of **2** were assigned as 1R*, 4R*, 5R*, 8S* and 9S*.

The *in vitro* anti-inflammatory effects of sesquiterpenoids **1** and **2** were tested. Caryophyllene **1** and **2** displayed inhibitory effects on the generation of superoxide anions (inhibition rates = 31.95% and 42.22%, respectively) and the release of elastase (inhibition rates = 51.64% and 42.10%, respectively) by human neutrophils at concentrations of 42.4 and 37.6 μM (10 μg/mL for Compounds **1** and **2**), respectively.

## 3. Experimental Section

### 3.1. General Experimental Procedures

Optical rotation values were measured with a Jasco P-1010 digital polarimeter (Japan Spectroscopic Corporation, Tokyo, Japan). IR spectra were obtained on a Varian Digilab FTS 1000 FT-IR spectrophotometer (Varian Inc., Palo Alto, CA, USA); peaks are reported in cm^−^^1^. NMR spectra were recorded on a Varian Mercury Plus 400 NMR spectrometer (Varian Inc., Palo Alto, CA, USA) using the residual CHCl_3_ signal (δ_H_ 7.26 ppm) as the internal standard for ^1^H NMR and CDCl_3_ (δ_C_ 77.1 ppm) for ^13^C NMR. Coupling constants (*J*) are given in Hz. ESIMS and HRESIMS were recorded using a Bruker 7 Tesla solariX FTMS system (Bruker, Bremen, Germany). Column chromatography was performed on silica gel (230–400 mesh, Merck, Darmstadt, Germany). TLC was carried out on precoated Kieselgel 60 F_254_ (0.25 mm, Merck, Darmstadt, Germany); spots were visualized by spraying with 10% H_2_SO_4_ solution followed by heating. Normal-phase HPLC (NP-HPLC) was performed using a system comprised of a Hitachi L-7110 pump (Hitachi Ltd., Tokyo, Japan), a Hitachi L-7455 photodiode array detector (Hitachi Ltd., Tokyo, Japan) and a Rheodyne 7725 injection port (Rheodyne LLC, Rohnert Park, CA, USA). A semi-preparative normal-phase column (Hibar 250 × 10 mm, LiChrospher Si 60, 5 μm, Merck, Darmstadt, Germany) was used for HPLC.

### 3.2. Animal Material

Specimens of the gorgonian coral, *Rumphella antipathies* (Nutting), were collected by hand using scuba equipment off the coast of Pingtung, Southern Taiwan. This organism was identified by comparison with previous descriptions [5]. A voucher specimen (Specimen No. NMMBA-TWGC-010) was deposited in the National Museum of Marine Biology and Aquarium, Taiwan.

### 3.3. Extraction and Isolation

Sliced bodies of the gorgonian *R. antipathies* (wet weight 402 g, dry weight 144 g) were extracted with a mixture of methanol (MeOH) and dichloromethane (CH_2_Cl_2_) (1:1) at room temperature. The extract was partitioned with ethyl acetate (EtOAc) and H_2_O. The EtOAc layer was separated by silica gel and eluted using *n*-hexane/EtOAc (stepwise, 25:1–pure EtOAc) to yield 29 fractions. Every fraction was checked using the ^1^H NMR spectra. Fractions 12 and 17 were re-purified by normal-phase HPLC (NP-HPLC) using a mixture of CH_2_Cl_2_ and EtOAc as the mobile phase to afford **2** (15.0 mg, 9:1) and **1** (1.0 mg, 15:1), respectively.

Rumphellol A (**1**): Colorless oil; 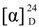 −55 (*c* 0.04, CHCl_3_); IR (neat) ν_max_ 3429, 1724 cm^−1^; ^1^H NMR (CDCl_3_, 400 MHz) and ^13^C NMR (CDCl_3_, 100 MHz) data, see Table 1; ESIMS *m*/*z* 237 [M + H]^+^; HRESIMS *m*/*z* 237.1836 (calcd. for C_15_H_24_O_2_ + H, 237.1849).

Rumphellol B (**2**): Colorless oil; 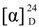 +12 (*c* 0.27, CHCl_3_); IR (neat) ν_max_ 3441 cm^−1^; ^1^H NMR(CDCl_3_, 400 MHz) and ^13^C NMR (CDCl_3_, 100 MHz) data, see Table 2; ESIMS *m*/*z* 289 [M + Na]^+^; HRESIMS *m*/*z* 289.2128 (calcd for C_17_H_30_O_2_ + Na, 289.2138).

### 3.4. Human Neutrophil Superoxide Anion Generation and Elastase Release

Human neutrophils were obtained by means of dextran sedimentation and Ficoll centrifugation. Superoxide anion generation was carried out according to the procedures described previously [25,26]. Briefly, superoxide anion production was assayed by monitoring the superoxide dismutase-inhibitable reduction of ferricytochrome *c*. Elastase release experiments were performed using MeO-Suc-Ala-Ala-Pro-Val-*p*-nitroanilide as the elastase substrate. DPI (diphenyleneiodonium) and elastatinal were used as reference compounds in the anti-inflammatory test of the inhibitory effects on the generation of superoxide anions (IC_50_ = 3.26 μM) and the release of elastase (IC_50_ = 60.0 μM) by human neutrophils in response to fMet-Leu-Phe/Cytochalastin B (FMLP/CB) respectively. In the *in vitro* anti-inflammatory bioassay, the inhibitory effects on the generation of superoxide anion and the release of elastase by activated neutrophils were used as indicators. At a concentration of 10 μg/mL, for the significant activity of pure compounds, an inhibition rate ≥50% is required (inhibition rate ≤ 10%, not active; 20% ≥ inhibition rate ≥ 10%, weakly anti-inflammatory; 50% ≥ inhibition rate ≥ 20%, modestly anti-inflammatory).

## 4. Conclusions

Only one previous study has focused on the chemical components of the gorgonian coral, *Rumphella aggregata* [27]. The use of organic extracts from gorgonians belonging to the *Rumphella* genus in ecology and for medical use has also been reported [28,29]. In continuing studies of new substances from marine invertebrates collected off the waters of Taiwan, two new caryophyllene-type sesquiterpenoids, rumphellols A and B (**1** and **2**), were isolated from the gorgonian coral, *Rumphella antipathies*. The structures of new sesquiterpenoids **1** and **2** were elucidated on the basis of spectroscopic methods, and these two compounds were found to display inhibitory effects on the generation of superoxide anions and the release of elastase by human neutrophils. The gorgonian coral, *Rumphella antipathies*, has been transplanted to culturing tanks located in the National Museum of Marine Biology and Aquarium, Taiwan, for extraction of additional natural products to establish a stable supply of bioactive material.
